# Comparing the effects of *GBA* variants and onset age on clinical features and progression in Parkinson's disease

**DOI:** 10.1111/cns.14387

**Published:** 2023-08-10

**Authors:** Jingru Ren, Xiaoyan Zhan, Hao Zhou, Zhiying Guo, Yi Xing, Hangxing Yin, Chen Xue, Jun Wu, Weiguo Liu

**Affiliations:** ^1^ Department of Neurology The Affiliated Brain Hospital of Nanjing Medical University Nanjing China; ^2^ Jiangsu Province Hospital of Traditional Chinese Medicine Nanjing China; ^3^ Department of Radiology The Affiliated Brain Hospital of Nanjing Medical University Nanjing China; ^4^ Department of Clinical Laboratory The Affiliated Brain Hospital of Nanjing Medical University Nanjing China

**Keywords:** age at onset, cognitive decline, *GBA*, motor impairment, Parkinson's disease

## Abstract

**Objective:**

Glucosylceramidase (*GBA*) variants and onset age significantly affect clinical phenotype and progression in Parkinson's disease (PD). The current study compared clinical characteristics at baseline and cognitive and motor progression over time among patients having *GBA*‐related PD (*GBA*‐PD), early‐onset idiopathic PD (early‐iPD), and late‐onset idiopathic PD (late‐iPD).

**Methods:**

We recruited 88 *GBA*‐PD, 167 early‐iPD, and 488 late‐iPD patients in this study. A subset of 50 *GBA*‐PD, 81 early‐iPD, and 223 late‐iPD patients was followed up at least once, with a 3.0‐year mean follow‐up time. Linear mixed‐effects models helped evaluate the rate of change in the Unified Parkinson's Disease Rating Scale motor and Montreal Cognitive Assessment scores.

**Results:**

At baseline, the *GBA*‐PD group showed more severe motor deficits and non‐motor symptoms (NMSs) than the early‐iPD group and more NMSs than the late‐iPD group. Moreover, the *GBA*‐PD group had more significant cognitive and motor progression, particularly bradykinesia and axial impairment, than the early‐iPD and late‐iPD groups at follow‐up. However, the early‐onset *GBA*‐PD (early‐*GBA*‐PD) group was similar to the late‐onset *GBA*‐PD (late‐*GBA*‐PD) group in baseline clinical features and cognitive and motor progression.

**Conclusion:**

*GBA*‐PD patients exhibited faster cognitive and motor deterioration than early‐iPD and late‐iPD patients. Thus, subtype classification based on genetic characteristics rather than age at onset could enhance the prediction of PD disease progression.

## Graphical abstract

*GBA*‐related Parkinson's disease (PD) patients exhibited faster cognitive and motor deterioration, particularly bradykinesia and axial impairment, than early‐onset idiopathic PD and late‐onset idiopathic PD patients. Subtype classification based on genetic characteristics rather than age at onset could enhance the prediction of PD disease progression.

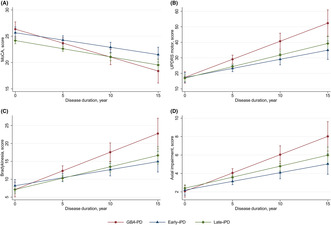

## 1 INTRODUCTION

Parkinson's disease (PD) is a chronic age‐related neurodegenerative disease having widespread motor and non‐motor symptoms.^1^ PD is characterized by the loss of dopaminergic neurons in the substantia nigra and alpha‐synuclein (α‐syn) protein aggregation.^2^ Heterozygous variants within the glucocerebrosidase (*GBA*; OMIM 606463) gene that encode the lysosomal enzyme β‐glucocerebrosidase (GCase) are the most common global genetic contributors to PD susceptibility.^3^, ^4^ Notably, ethnic heterogeneity could be observed in *GBA* variant frequency, estimated at 10%–31% in the Ashkenazi Jewish (AJ), 3%–12% in the non‐AJ North American, and 2%–11% in the Chinese populations.^5^, ^6^, ^7^, ^8^

Overall, characteristic *GBA*‐related PD (*GBA*‐PD) patients have earlier onset with more severe motor impairment, frequent non‐motor symptoms (NMSs, particularly cognitive impairment, rapid eye movement sleep disorder, and autonomic dysfunction), aggressive disease progression, and lower survival rate than idiopathic PD (iPD) patients.^8^, ^9^, ^10^, ^11^, ^12^ Interestingly, the onset age also significantly affects the clinical phenotype and disease progression of PD.^13^, ^14^, ^15^ Early‐onset iPD (early‐iPD) patients generally possess a lower cognitive deficit risk, a higher levodopa‐associated dyskinesia prevalence, and slower disease progression.^16^, ^17^ However, late‐onset iPD (late‐iPD) patients exhibit more significant dopaminergic impairment, and severe motor and non‐motor symptoms, with faster disease progression.^18^ Thus, both *GBA*‐PD and late‐iPD could lead to a malignant disease course.

The existence of differences between *GBA*‐PD and late‐iPD based on disease progression remains unknown. Recently, one study based on the Parkinson's Progression Markers Initiative (PPMI) dataset revealed that *GBA*‐PD had a more severe motor and cognitive decline and faster global cognitive deterioration than early‐iPD, but similar to late‐iPD.^19^ However, only a few studies have investigated the clinical manifestations and disease progression associated with *GBA*‐PD, early‐iPD, and late‐iPD. Moreover, the impact of onset age on cognitive and motor impairment in *GBA*‐PD patients has not been elucidated. The research gaps were mitigated by performing a detailed comparison of clinical characteristics at baseline and cognitive and motor progression over time between *GBA*‐PD, early‐iPD, and late‐iPD groups and between those groups of early‐onset *GBA*‐PD (early‐*GBA*‐PD) and late‐onset *GBA*‐PD (late‐*GBA*‐PD) within a large Chinese cohort.

## 2 METHODS

### 2.1 Participants

We conducted a comprehensive longitudinal study involving a large cohort of Chinese PD patients between 2012 and 2023 at the Department of Neurology, the Affiliated Brain Hospital of Nanjing Medical University. All the PD patients satisfying the UK Parkinson's Disease Society Brain Bank clinical diagnostic criteria^20^ underwent *GBA* genetic testing. Only those patients were excluded who had atypical or secondary parkinsonism disorders, a history of severe chronic diseases, including renal or heart failure, clinically significant lesions on routine brain magnetic resonance imaging (MRI), and difficulty obtaining demographic and clinical data. If available, PD patients satisfying the inclusion and exclusion criteria were invited to participate in the annual follow‐up visits at our institution.

The Medical Ethics Committee of the Affiliated Brain Hospital of Nanjing Medical University provided ethical approval for this study. Written informed consent was obtained from all the patients before study participation.

### 2.2 Clinical evaluation

Face‐to‐face interviews were conducted to collect detailed demographic and clinical data from all PD patients at baseline and each follow‐up visit. Based on the first onset age of PD‐related motor symptoms (i.e., rest tremor, bradykinesia, or rigidity), the *GBA*‐PD and iPD patients were divided into two groups: early‐onset (onset age ≤50 years) and late‐onset (onset age >50 years). Dopaminergic drug information was recorded, and an established method was used to determine the levodopa equivalent daily dose (LEDD) for all drugs.^21^ The Unified Parkinson's Disease Rating Scale (UPDRS) Part II and III and modified Hoehn–Yahr (H–Y) stages helped reflect the activities of daily living (ADL), motor impairment, and the disease severity, respectively. The specific motor characteristics of PD patients were evaluated by summarizing the related UPDRS III items: tremor (items 20 and 21), rigidity (item 22), bradykinesia (items 23–26 and 31), and axial impairment (items 27–30). The cognitive, emotional, and sleep status were measured using the Mini‐Mental State Examination (MMSE) and Montreal Cognitive Assessment (MoCA), Hamilton Depression Scale (HAMD) and Hamilton Anxiety Scale (HAMA), and the Parkinson's Disease Sleep Scale (PDSS). If the education years in PD patients were ≤12 years, the total MoCA (if <30) score was increased by one point.^22^ The presence of NMSs was determined using the Non‐Motor Symptoms Questionnaire (NMSQuest), divided into nine domains: cardiovascular, sleep, mood/cognitive, perception/hallucinations, attention/memory, gastrointestinal, urinary, sexual function, and miscellaneous.^23^, ^24^

### 2.3 Molecular genetic analysis of *GBA* variants

*GBA* gene avoided contamination with the adjacent pseudogene *GBAP1* through long‐range polymerase chain reaction (LR‐PCR) and next‐generation sequencing (NGS).^8^ Based on the *GBA* variant carrier, PD patients were divided into *GBA*‐PD and iPD groups. Furthermore, *GBA* variants possessed five grade categories: mild (causing Gaucher disease [GD] type 1), severe (causing GD type 2 or 3), risk (related to higher PD risk but not significant to GD), complex (≥2 *GBA* variants), and unknown.^25^

### 2.4 Statistical analysis

The Kolmogorov–Smirnov (*n* > 50) or the Shapiro–Wilk tests (*n* ≤ 50) helped assess quantitative variables, which are expressed as mean and standard deviation (SD). Moreover, qualitative variables could be expressed as frequency and proportion. We compared the baseline demographic among the *GBA*‐PD, early‐iPD, and late‐iPD groups and among the early‐*GBA*‐PD, late‐*GBA*‐PD, early‐iPD, and late‐iPD groups. The analysis of variance (ANOVA) helped compare the quantitative variables obeying the normal distribution. Moreover, not normally distributed quantitative variables were compared with the Kruskal–Wallis *H*‐test. The chi‐squared test helped analyze the qualitative variables. The baseline clinical characteristics were compared using a general linear model (GLM), adjusting for confounders in Table 1. For multiple comparisons, all the analyses underwent Bonferroni correction. The logistic regression was adjusted for sex and disease duration to compare nine domains of NMSQuest among the *GBA*‐PD, early‐iPD, and late‐iPD groups.

**TABLE 1:** Demographic and clinical characteristics of the PD patients at baseline.

Variable	*GBA*‐PD (*n* = 88)	Early‐*GBA*‐PD (*n* = 39)	Late‐*GBA*‐PD (*n* = 49)	Early‐iPD (*n* = 167)	Late‐iPD (*n* = 488)	*P*_1_	*P*_2_
Sex, male (%)	46 (52.3)	17 (43.6)	29 (59.2)	101 (60.5)	260 (53.3)	0.240	0.173
Age at onset (years)	52.4 ± 10.0	43.7 ± 5.6	59.3 ± 6.7	44.1 ± 6.5	60.9 ± 6.3	**<0.001** ^**a,b,c**^	**<0.001** ^**d,f,g,i**^
Age (years)	56.3 ± 9.5	48.9 ± 6.4	62.1 ± 7.3	50.0 ± 7.8	64.3 ± 6.5	**<0.001** ^**a,b,c**^	**<0.001** ^**d,f,g,i**^
Education (years)	9.2 ± 4.0	9.4 ± 3.8	9.1 ± 4.2	10.3 ± 3.8	10.1 ± 4.2	0.121	0.238
Disease duration (years)	3.9 ± 3.7	5.1 ± 4.5	2.9 ± 2.5	5.9 ± 5.8	3.4 ± 3.1	**<0.001** ^**a,c**^	**<0.001** ^**g,i**^
LEDD (mg/day)^§^	325.1 ± 293.0	380.1 ± 336.2	281.4 ± 248.3	375.4 ± 392.7	293.3 ± 290.9	0.445	0.651
UPDRS ADL score^†^	11.1 ± 6.8	11.9 ± 7.5	10.4 ± 6.2	9.7 ± 5.1	9.7 ± 5.7	**<0.001** ^**a,c**^	**<0.001** ^**e,g,i**^
UPDRS motor score^†^	27.0 ± 14.7	31.3 ± 16.0	23.6 ± 12.8	23.7 ± 13.2	23.7 ± 12.6	**0.002** ^**a,c**^	**0.001** ^**e,f**^
UPDRS motor subscores^†^							
Tremor	3.6 ± 3.1	3.4 ± 3.0	3.7 ± 3.2	3.5 ± 3.2	3.3 ± 3.2	0.545	0.390
Rigidity	6.1 ± 4.8	7.5 ± 4.7	5.0 ± 4.7	5.4 ± 4.3	5.0 ± 3.8	0.070	**0.009** ^**e,f**^
Bradykinesia	11.7 ± 7.6	14.4 ± 8.3	9.6 ± 6.4	10.2 ± 6.7	10.5 ± 6.6	**0.018** ^**a**^	**0.002** ^**e,f**^
Axial impairment	3.7 ± 2.6	4.0 ± 2.9	3.4 ± 2.3	3.2 ± 1.9	3.4 ± 2.3	**0.001** ^**a,c**^	**0.004** ^**i**^
H–Y stage^†^	2.0 ± 0.8	2.1 ± 0.9	1.8 ± 0.7	1.8 ± 0.7	1.9 ± 0.7	**<0.001** ^**a,c**^	**<0.001** ^**e,i**^
MMSE score^¶^	26.1 ± 4.5	26.4 ± 4.6	25.8 ± 4.4	27.4 ± 3.2	26.5 ± 3.7	**<0.001** ^**a,c**^	**<0.001** ^**g,i**^
MoCA score^¶^	22.6 ± 5.6	22.7 ± 6.3	22.4 ± 5.1	23.8 ± 4.6	22.1 ± 4.9	**<0.001** ^**c**^	**<0.001** ^**i**^
HAMD score^§^	11.9 ± 8.7	12.1 ± 9.1	11.7 ± 8.4	10.5 ± 7.1	10.1 ± 7.5	0.133	0.236
HAMA score^§^	9.0 ± 7.3	8.7 ± 7.0	9.2 ± 7.7	6.9 ± 5.0	7.2 ± 5.3	**0.004** ^**a,b**^	**0.006** ^**g,h**^
PDSS score^§^	109.0 ± 28.5	110.8 ± 29.2	107.5 ± 28.0	119.9 ± 22.7	116.8 ± 23.6	**<0.001** ^**a,b,c**^	**<0.001** ^**g,h,i**^
NMSQuest score^§^	10.9 ± 6.2	10.9 ± 6.2	10.8 ± 6.4	7.7 ± 4.4	9.1 ± 4.8	**<0.001** ^**a,b,c**^	**<0.001** ^**e,g,h,i**^

*Note*: Data are represented as mean ± SD or *n* (%). Group comparisons were determined using the chi‐squared test, Kruskal–Wallis *H*‐test, or general linear model (GLM). These were followed by post hoc analysis using the Bonferroni adjustment. *P*_1_ indicates the *p*‐value of comparison among *GBA*‐PD, early‐iPD, and late‐iPD groups. *P*_2_ depicts the *p*‐value of comparison among early‐*GBA*‐PD, late‐*GBA*‐PD, early‐iPD, and late‐iPD groups. Bold indicates statistically significant differences (*p* < 0.05). Post hoc analyses: a: *GBA*‐PD versus early‐iPD; b: *GBA*‐PD versus late‐iPD; c: late‐iPD versus early‐iPD; d: early‐*GBA*‐PD versus late‐*GBA*‐PD; e: early‐*GBA*‐PD versus early‐iPD; f: early‐*GBA*‐PD versus late‐iPD; g: late‐*GBA*‐PD versus early‐iPD; h: late‐*GBA*‐PD versus late‐iPD groups; i: early‐iPD versus late‐iPD.

Abbreviations: ADL, activities of daily living; early‐*GBA*‐PD, early‐onset *GBA*‐PD; early‐iPD, early‐onset iPD; *GBA*‐PD, *GBA*‐related PD; HAMD, Hamilton Depression Scale; HAMA, Hamilton Anxiety Scale; H–Y, Hoehn–Yahr; iPD, idiopathic PD; late‐*GBA*‐PD, late‐onset *GBA*‐PD; late‐iPD, late‐onset iPD; LEDD, levodopa equivalent daily dose; MMSE, Mini‐Mental State Examination; MoCA, Montreal Cognitive Assessment; NMSQuest, Non‐Motor Symptoms Questionnaire; PD, Parkinson's disease; PDSS, Parkinson's Disease Sleep Scale; UPDRS, Unified Parkinson's Disease Rating Scale.

^§^ Adjusted for sex and disease duration.

^†^ Adjusted for sex, disease duration, and LEDD.

^¶^ Adjusted for sex, disease duration, and years of formal education.

Linear mixed‐effects models were used with PD duration as the timescale to compare the rate of change in UPDRS motor and MoCA scores among the groups. The linear mixed effect model showed the following advantages: (1) available data usage during the follow‐up period, (2) accommodating unbalanced data due to missing data points, dropout, uneven visit interval, etc., and (3) considering the correlation between repeated measurements between individuals so that this model can clarify the longitudinal marker trajectory. Time, group, and interaction were the fixed effects, adjusting for sex, LEDD, or education. All the models had random intercepts of the IDs of patients and random time slopes. Margins and marginsplot were used in Stata to plot marginal motor and cognitive progression predictions. IBM SPSS Statistics version 27.0 or Stata version 17.0 was used for statistical analyses. Two‐sided *p* < 0.05 were considered statistically significant.

## 3 RESULTS

### 3.1 Baseline demographic and clinical characteristics

Demographic and clinical characteristics of 743 PD patients (88 *GBA*‐PD, 167 early‐iPD, and 488 late‐iPD) are represented in Table 1. Around 354 PD patients (50 *GBA*‐PD, 81 early‐iPD, and 223 late‐iPD) were followed up at least once, with a mean follow‐up time of 3.0 years. Table S1 summarizes the details concerning all the identified *GBA* variants.

**FIGURE 1:**
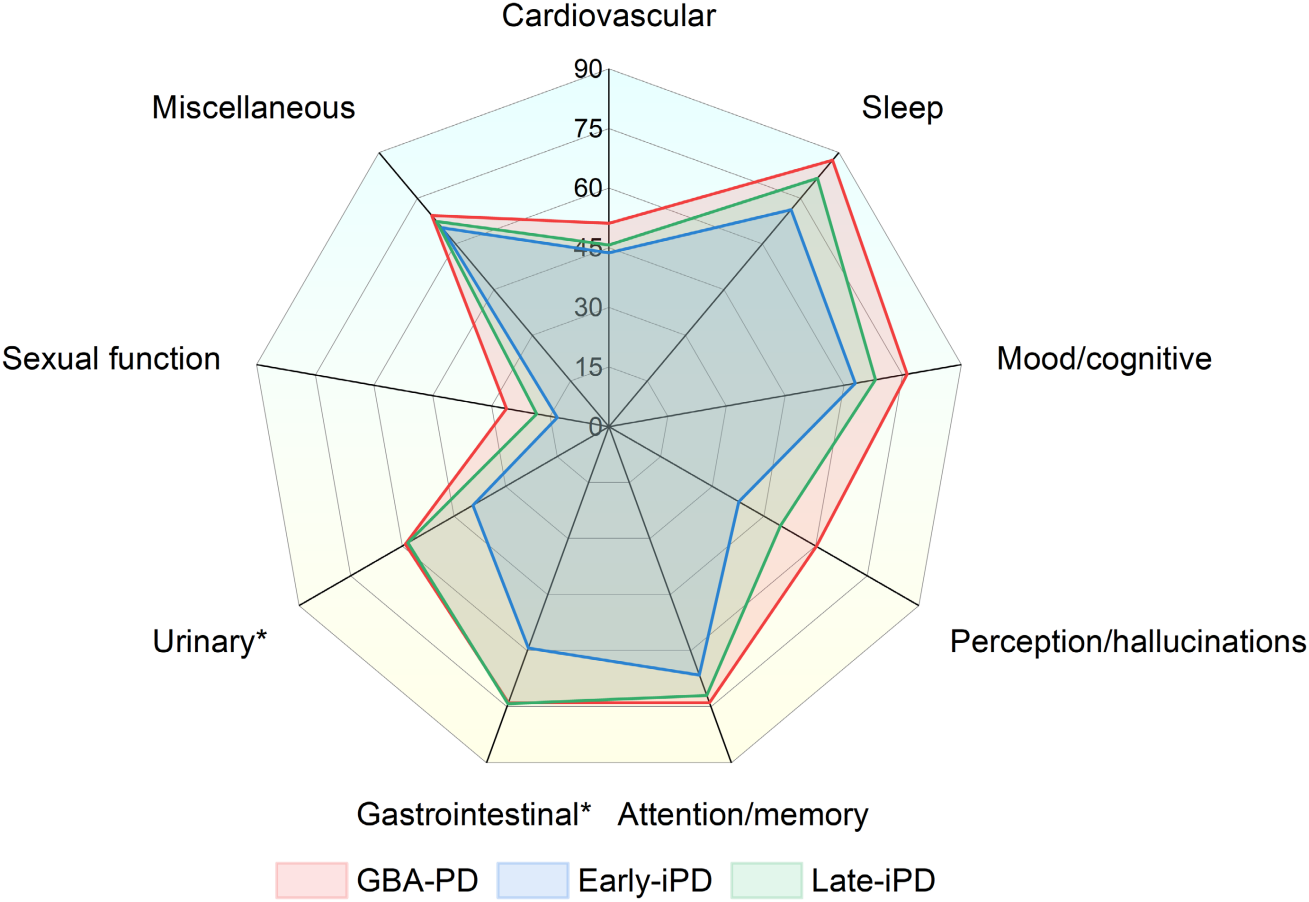
Frequency of NMSs domain deficits among the *GBA*‐PD, early‐iPD, and late‐iPD groups. Groups were compared using logistic regression and adjusted for sex and disease duration. *p < 0.05. early‐iPD, early‐onset iPD; *GBA*‐PD, *GBA*‐related PD; iPD, idiopathic PD; late‐iPD, late‐onset iPD; NMSs, non‐motor symptoms; PD, Parkinson's disease.

No significant difference was observed in sex, education, LEDD, tremor, rigidity, and HAMD among the *GBA*‐PD, early‐iPD, and late‐iPD groups. At the same time, significant differences were seen in age at onset, age, disease duration, UPDRS ADL, UPDRS motor, bradykinesia, axial impairment, H–Y stage, MMSE, MoCA, HAMA, PDSS, and NMSQuest. In the post hoc analysis, the *GBA*‐PD group had a later age at onset and age, shorter disease duration, highly severe scores of UPDRS ADL, UPDRS motor, bradykinesia, axial impairment, H–Y stage, MMSE, HAMA, PDSS, and NMSQuest, compared to early‐iPD. The late‐iPD group had a later age at onset and age, shorter disease duration, very severe scores of UPDRS ADL, UPDRS motor, axial impairment, H–Y stage, MMSE, MoCA, PDSS, and NMSQuest compared to early‐iPD. Additionally, *GBA*‐PD had an earlier age at onset and age, with more severe scores of HAMA, PDSS, and NMSQuest than late‐iPD (Table 1). Furthermore, based on NMSQuest, the frequency of nine domains was compared among the *GBA*‐PD, early‐iPD, and late‐iPD groups (Figure 1; Table S2). *GBA*‐PD and late‐iPD patients showed a higher gastrointestinal and urinary symptom prevalence than early‐iPD patients.

Significant differences could be observed among the early‐*GBA*‐PD, late‐*GBA*‐PD, early‐iPD, and late‐iPD groups regarding age at onset, age, disease duration, UPDRS ADL, UPDRS motor, rigidity, bradykinesia, axial impairment, H–Y stage, MMSE, MoCA, HAMA, PDSS, and NMSQuest. In the post hoc analysis, the early‐*GBA*‐PD group had an earlier onset age and younger baseline age than the late‐*GBA*‐PD group. Early‐*GBA*‐PD patients possessed more severe scores such as UPDRS ADL, UPDRS motor, rigidity, bradykinesia, H–Y stage, and NMSQuest than early‐iPD patients. In contrast, late‐*GBA*‐PD patients exhibited more severe HAMA, PDSS, and NMSQuest scores than late‐iPD patients (Table 1).

### 3.2 Cognitive progression

**FIGURE 2:**
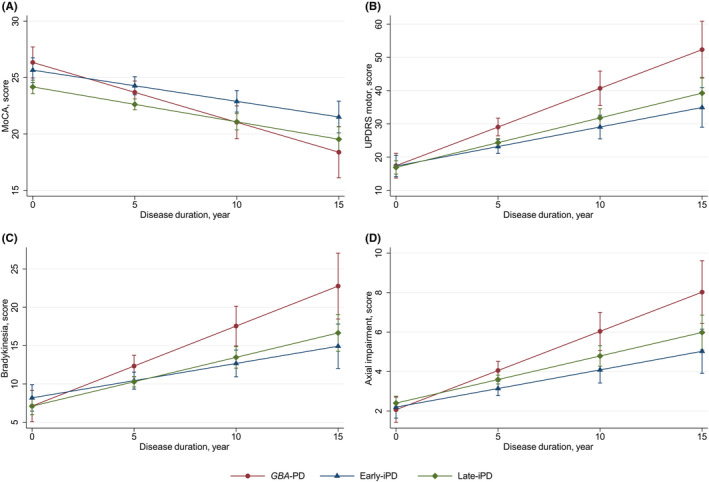
The longitudinal trajectories of mean MoCA and UPDRS motor scores among the *GBA*‐PD, early‐iPD, and late‐iPD groups. early‐iPD, early‐onset iPD; *GBA*‐PD, *GBA*‐related PD; iPD, idiopathic PD; late‐iPD, late‐onset iPD; MoCA, Montreal Cognitive Assessment; PD, Parkinson's disease; UPDRS, Unified Parkinson's Disease Rating Scale.

Linear mixed‐effects models helped compare the rate of change in MoCA score while adjusting for sex and education (Table 2; Figure 2A). The estimated (standard error, SE) progression rates for the MoCA score were −0.53 (0.10) points/year for *GBA*‐PD, −0.28 (0.06) points/year for early‐iPD, and −0.31 (0.05) points/year for late‐iPD. A worse cognitive decline rate was observed in *GBA*‐PD than in early‐iPD (*B* [SE], −0.25 [0.12] points/year; *p* = 0.035) and late‐iPD (*B* [SE], −0.22 [0.11] points/year; *p* = 0.047) patients. The cognitive progression rate was faster in severe *GBA*‐PD patients than those in early‐iPD (*B* [SE], −0.37 [0.17] points/year; *p* = 0.029) and late‐iPD (*B* [SE], −0.34 [0.16] points/year; *p* = 0.037) groups. However, no significant difference was observed in the cognitive progression rate among the early‐*GBA*‐PD, late‐*GBA*‐PD, early‐iPD, and late‐iPD participants.

**TABLE 2:** Comparing the rate of change in MoCA and UPDRS motor scores among the groups.

Characteristic	MoCA^a^		UPDRS motor^b^	
	*B* (SE)	*p*	*B* (SE)	*p*
Among the *GBA*‐PD, early‐iPD, and late‐iPD groups				
PD slope, points/year				
*GBA*‐PD	−0.53 (0.10)	<0.001	2.33 (0.37)	<0.001
Early‐iPD	−0.28 (0.06)	<0.001	1.18 (0.27)	<0.001
Late‐iPD	−0.31 (0.05)	<0.001	1.49 (0.21)	<0.001
PD slope differences between groups, point/year				
*GBA*‐PD versus Early‐iPD	−0.25 (0.12)	0.035	1.15 (0.43)	0.007
*GBA*‐PD versus Late‐iPD	−0.22 (0.11)	0.047	0.84 (0.39)	0.030
Late‐iPD versus Early‐iPD	−0.03 (0.08)	0.685	0.31 (0.30)	0.297
Among the severe *GBA*‐PD, early‐iPD, and late‐iPD groups				
PD slope, points/year				
Severe *GBA*‐PD	−0.65 (0.15)	<0.001	2.68 (0.55)	<0.001
Early‐iPD	−0.28 (0.07)	<0.001	1.17 (0.26)	<0.001
Late‐iPD	−0.31 (0.05)	<0.001	1.47 (0.20)	<0.001
PD slope differences between groups, point/year				
Severe *GBA*‐PD versus Early‐iPD	−0.37 (0.17)	0.029	1.50 (0.58)	0.009
Severe *GBA*‐PD versus Late‐iPD	−0.34 (0.16)	0.037	1.21 (0.55)	0.028
Late‐iPD versus Early‐iPD	−0.03 (0.08)	0.716	0.30 (0.28)	0.295
Among the early‐*GBA*‐PD, late‐*GBA*‐PD, early‐iPD, and late‐iPD groups				
PD slope, points/year				
Early‐*GBA*‐PD	−0.54 (0.16)	<0.001	2.37 (0.54)	<0.001
Late‐*GBA*‐PD	−0.55 (0.14)	<0.001	2.23 (0.49)	<0.001
Early‐iPD	−0.28 (0.06)	<0.001	1.18 (0.27)	<0.001
Late‐iPD	−0.31 (0.05)	<0.001	1.49 (0.21)	<0.001
PD slope differences between groups, point/year				
Early‐*GBA*‐PD versus Late‐*GBA*‐PD	0.01 (0.21)	0.975	0.14 (0.70)	0.839
Early‐*GBA*‐PD versus Early‐iPD	−0.27 (0.17)	0.112	1.19 (0.58)	0.039
Late‐*GBA*‐PD versus Early‐iPD	−0.27 (0.15)	0.070	1.05 (0.53)	0.050
Late‐iPD versus Early‐iPD	−0.03 (0.08)	0.687	0.32 (0.30)	0.295
Early‐*GBA*‐PD versus Late‐iPD	−0.23 (0.16)	0.147	0.88 (0.55)	0.108
Late‐*GBA*‐PD versus Late‐iPD	−0.24 (0.14)	0.094	0.73 (0.50)	0.143

*Note*: UPDRS motor and MoCA scores are available for 50 *GBA*‐PD, 81 early‐iPD, and 223 late‐iPD patients.

Abbreviations: early‐*GBA*‐PD, early‐onset *GBA*‐PD; early‐iPD, early‐onset iPD; *GBA*‐PD, *GBA*‐related PD; iPD, idiopathic PD; late‐*GBA*‐PD, late‐onset *GBA*‐PD; late‐iPD, late‐onset iPD; MoCA, Montreal Cognitive Assessment; PD, Parkinson's disease; SE, standard error; UPDRS, Unified Parkinson's Disease Rating Scale.

^a^ Model adjusted for sex and education.

^b^ Model adjusted for sex and levodopa equivalent daily dose at visits.

### 3.3 Motor progression

Linear mixed‐effects models compared the rate of change in UPDRS motor scores while adjusting for sex and LEDD during visits (Table 2; Figure 2B). The estimated (SE) progression rates for the UPDRS motor score were 2.33 (0.37) points/year for *GBA*‐PD, 1.18 (0.27) points/year for early‐iPD, and 1.49 (0.21) points/year for late‐iPD. A faster motor progression rate was present for the *GBA*‐PD group compared with the early‐iPD group (*B* [SE], 1.15 [0.43] points/year; *p* = 0.007) and late‐iPD group (*B* [SE], 0.84 [0.39] points/year; *p* = 0.030). Similarly, severe *GBA*‐PD had a worse motor decline rate than early‐iPD (*B* [SE], 1.50 [0.58] points/year; *p* = 0.009) and late‐iPD (*B* [SE], 1.21 [0.55] points/year; *p* = 0.028) patients. Additionally, the motor progression rate was more severe in early‐*GBA*‐PD patients than those in early‐iPD (*B* [SE], 1.19 [0.58] points/year; *p* = 0.039). However, no difference in motor progression rate was observed between early‐*GBA*‐PD and late‐*GBA*‐PD, late‐iPD and early‐iPD, and late‐*GBA*‐PD and late‐iPD participants.

Subanalyses examining the changes in specific motor domains indicated that motor progression differences among *GBA*‐PD, early‐iPD, and late‐iPD groups were primarily due to changes in bradykinesia and axial impairment scores (Table 3; Figure 2C,D). The estimated (SE) progression rates for the bradykinesia score were 1.04 (0.19) points/year for *GBA*‐PD, 0.45 (0.14) points/year for early‐iPD, and 0.64 (0.11) points/year for late‐iPD. Moreover, the estimated (SE) progression rates for the axial impairment score were 0.40 (0.07) points/year for *GBA*‐PD, 0.19 (0.05) points/year for early‐iPD, and 0.24 (0.04) points/year for late‐iPD. A faster progression rate for bradykinesia could be observed for the *GBA*‐PD group compared with the early‐iPD group (*B* [SE], 0.59 [0.22] points/year; *p* = 0.006) and the late‐iPD group (*B* [SE], 0.41 [0.20] points/year; *p* = 0.040). Similarly, a faster progression rate of axial impairment for the *GBA*‐PD group was observed compared with the early‐iPD group (*B* [SE], 0.21 [0.08] points/year; *p* = 0.009) and late‐iPD group (*B* [SE], 0.16 [0.07] points/year; *p* = 0.027).

**TABLE 3:** Comparing the rate of change in UPDRS motor subscores among the *GBA*‐PD, early‐iPD, and late‐iPD groups.

Characteristic	Tremor		Rigidity		Bradykinesia		Axial impairment	
	*B* (SE)	*p*	*B* (SE)	*p*	*B* (SE)	*p*	*B* (SE)	*p*
PD slope, points/year								
*GBA*‐PD	0.20 (0.09)	0.027	0.47 (0.13)	<0.001	1.04 (0.19)	<0.001	0.40 (0.07)	<0.001
Early‐iPD	0.09 (0.07)	0.172	0.30 (0.09)	0.001	0.45 (0.14)	0.001	0.19 (0.05)	<0.001
Late‐iPD	0.09 (0.05)	0.071	0.36 (0.07)	<0.001	0.64 (0.11)	<0.001	0.24 (0.04)	<0.001
PD slope differences between groups, point/year								
*GBA*‐PD versus Early‐iPD	0.11 (0.10)	0.288	0.17 (0.15)	0.242	0.59 (0.22)	0.006	0.21 (0.08)	0.009
*GBA*‐PD versus Late‐iPD	0.11 (0.09)	0.249	0.11 (0.13)	0.408	0.41 (0.20)	0.040	0.16 (0.07)	0.027
Late‐iPD versus Early‐iPD	0.00 (0.07)	0.972	0.06 (0.10)	0.544	0.19 (0.15)	0.215	0.05 (0.06)	0.377

*Note*: Models adjusted for sex and levodopa equivalent daily dose at visits.

Abbreviations: early‐iPD, early‐onset iPD; *GBA*‐PD, *GBA*‐related PD; iPD, idiopathic PD; late‐iPD, late‐onset iPD; PD, Parkinson's disease; SE, standard error; UPDRS, Unified Parkinson's Disease Rating Scale.

## 4 DISCUSSION

The current study combined cross‐sectional and longitudinal designs to comprehensively assess the clinical features and cognitive and motor progression in a large cohort of Chinese *GBA*‐PD, early‐iPD, and late‐iPD patients. At baseline, the *GBA*‐PD group showed a more severe motor impairment and NMSs than the early‐iPD group and more NMSs than the late‐iPD group. Furthermore, the *GBA*‐PD patients had more pronounced cognitive and motor decline progression, particularly bradykinesia and axial impairment, than early‐iPD and late‐iPD patients. Importantly, baseline clinical features and disease progression were similar between late‐*GBA*‐PD and early‐*GBA*‐PD.

Recently, PD subtype identification has been an important research priority.^26^ Subtype identification can elevate the understanding of pathophysiological mechanisms, overall PD disease progression prediction, and ultimately the development of tailored treatment paradigms. Patients with the same causative genes and genetic risk factors could have similar underlying pathogenesis. This minimizes heterogeneity in clinical trials; thus, genetic features are increasingly utilized in PD subtype classification.^27^ PD patients with specific genetic variants (e.g., *GBA*, *LRRK2*, or *Parkin*) demonstrate distinct disease progression profiles. Among PD patients, disease progression is faster in *GBA* carriers but slower in *LRRK2* or *Parkin* carriers,^28^, ^29^, ^30^ increasing *GBA*‐PD research. In addition, age is crucial in the functional decline of dopaminergic neurons and PD development.^31^, ^32^ Traditionally, early‐iPD and late‐iPD exhibit distinct clinical features and disease progression depending on PD subtypes using the onset age as a single categorical variable. More rapid disease progression occurs in late‐iPD, which is slower in early‐iPD.^33^ Therefore, analyzing the differences between *GBA*‐PD, early‐iPD, and late‐iPD is essential for better stratifying PD subtypes to predict disease progression.

*GBA*‐PD patients at baseline experienced more severe motor impairment and NMSs than early‐iPD and more NMSs than late‐iPD patients. This could be attributed to the correlation between the severity of Lewy body pathology and clinical symptoms. The autopsy pathology of *GBA*‐PD patients was more extensive with diffused neocortical Lewy body type pathology than iPD patients.^5^ Experimental data validated the bidirectional pathogenic loop between GCase and α‐syn.^34^ Specifically, GCase function loss due to *GBA* variants impairs lysosomal α‐syn degradation. This causes α‐syn accumulation, while aggregated α‐syn inhibits GCase lysosomal activity. *GBA*‐PD patients may simultaneously satisfy the conditions of this positive feedback loop, forming a self‐propagating mechanism. Furthermore, recent studies with the PPMI dataset observed that *GBA*‐PD patients had lower striatal and standard uptake value ratios (SUVr) of extra‐striatal dopamine transporter (DAT), and had higher free water values in the posterior substantia nigra at baseline than in early‐iPD and late‐iPD groups.^19^, ^35^

Previously, we reported the impact of *GBA* variants on motor and cognitive impairment in PD patients. *GBA*‐PD had faster motor and cognitive progression than iPD, particularly in bradykinesia, axial impairment, and visuospatial/executive function.^9^ This study compared *GBA*‐PD with iPD stratified by the age of onset and observed faster cognitive and motor progression, particularly bradykinesia and axial impairment, in *GBA*‐PD patients than in early‐iPD and late‐iPD patients. However, a recent study in a subset of 168 PD cases revealed that the *GBA*‐PD group experienced significantly faster overall cognitive deterioration than the early‐iPD group.^19^ This difference may be because our study used a relatively large sample size of 354 PD patients for follow‐up. In a previous study, *GBA*‐PD and late‐iPD patients lost more than 1 MoCA point per year while assessing clinical progression using two different follow‐up points.^19^ However, in our study, which included all the available follow‐up data for analysis, *GBA*‐PD and late‐iPD lost 0.53 and 0.31 MoCA points yearly. Therefore, comprehensive elucidation of the differences between *GBA*‐PD, early‐iPD, and late‐iPD patients in more large longitudinal cohorts is necessary.

PD classification using the age of onset has been an attractive subtyping solution due to its ease of use in clinical settings.^36^ The severity of motor and non‐motor impairment, striatal binding damage, and cerebrospinal fluid biomarker levels in PD patients increase with the age of onset.^18^ Currently, there is a lack of research on the impact of onset age on cognitive and motor impairment in *GBA*‐PD patients. For the first time, our study revealed that late‐*GBA*‐PD and early‐*GBA*‐PD share similar baseline clinical features and disease progression over time, indicating that *GBA* has a significant role relative to aging within accelerating neurodegenerative disease processes.

The strengths of the current research are: (1) providing a novel comparative distinction between clinical characteristics and disease progression within the same large cohort of *GBA*‐PD, early‐iPD, and late‐iPD participants; (2) revealing for the first time that late‐*GBA*‐PD and early‐*GBA*‐PD patients have similar phenotypic features and disease progression depending on clinical data at baseline and follow‐up. However, many study limitations should also be considered. First, although the PD patients underwent complete *GBA* gene testing and avoided pseudogene interference, common PD‐related genes such as *LRRK2* and *Parkin* were not examined, which could affect the results. Therefore, excluding these common PD‐related genes is necessary, especially among early‐onset PD patients. Second, the number of PD subjects was relatively large. However, the average follow‐up time of 3.0 years should be extended, supporting a longer follow‐up duration. The clinical trajectory of Chinese PD patients will be continually monitored, and more patients will be recruited. In addition, follow‐up of *GBA*‐PD patients will be focused on this prospective cohort, hopefully providing the best data for *GBA*‐PD disease progression.

## 5 CONCLUSION

Our study combines cross‐sectional and longitudinal follow‐up to assess the clinical characteristics and disease progression within a large cohort of Chinese *GBA*‐PD, early‐iPD, and late‐iPD patients. *GBA*‐PD patients had more severe clinical manifestations and faster cognitive with motor progression than early‐iPD and late‐iPD patients. Notably, the late‐*GBA*‐PD and early‐*GBA*‐PD groups had similar clinical phenotypes and progression. These findings depict that subtype classification depending on genetic characteristics and not onset age would help predict prognosis and facilitate clinical trials of potentially disease‐modifying therapies.

## AUTHOR CONTRIBUTIONS

WL organized the project and critically revised the manuscript. JR organized the project, drafted the preliminary manuscript, collected data, and performed statistical analysis. XZ organized the project, collected data, and performed statistical analysis. HZ, ZG, YX, and HY collected data. CX and JW critiqued the statistical analysis and interpreted the data. All authors contributed to the article and approved the submitted manuscript.

## FUNDING INFORMATION

Science and Technology Development Project of Traditional Chinese Medicine in Jiangsu Province, Grant/Award Number: 2020ZX17; National Key Research and Development Program of China, Grant/Award Number: 2017YFC1310302 and 2016YFC1306600; National Natural Science Foundation of China, Grant/Award Number: 81571348; and Science and Technology Program of Jiangsu Province, Grant/Award Number: BE2019611.

## CONFLICT OF INTEREST STATEMENT

The authors declare no conflict of interest.

## Supporting information


Tables S1–S2.

## Data Availability

The original data of this study can be obtained from the corresponding author upon reasonable request.
