# Supplementary material for: Comparing the effects of GBA variants and onset age on clinical features and progression in Parkinson's disease
Source: CNS Neurosci Ther. 2023 Aug 10;30(2):e14387. doi: 10.1111/cns.14387 (PMC10848098; doi:10.1111/cns.14387)
Supplement: Supplementary file 1 — Tables S1–S2. [file CNS-30-e14387-s001.docx]

**Supplementary Materials**

**Supplementary Table**

**Table S1.** *GBA* variants identified among *GBA*-PD patients

**Table S2.** NMSs classified by domain within the *GBA*-PD, early-iPD, and late-iPD groups

**Table S1.** *GBA* variants identified among *GBA*-PD patients

| **Cases** | **Early-onset** | **Late-onset** | **Allele name**  **(HGVS)** | **Allele name**  **(traditional)** | **Amino acid**  **change** | **Nucleotide**  **change** | **Exon** | **Class of**  **variant** | **RS (dbSNP)** |
| --- | --- | --- | --- | --- | --- | --- | --- | --- | --- |
| 3 | 0 | 3 | L303I | L264I | p.Leu303Ile | c.907C>A | 8 | mild | rs1296507371 |
| 1 | 1 | 0 | D438H | D399H | p.Asp438His | c.1312G>C | 10 | mild | - |
| 1 | 1 | 0 | F76V | F37V | p.Phe76Val | c.226T>G | 4 | mild | - |
| 1 | 1 | 0 | V414L | V375L | p.Val414Leu | c.1240G>C | 10 | mild | rs398123528 |
| 1 | 1 | 0 | V499M | V460M | p.Val499Met | c.1495G>A | 11 | mild | rs369068553 |
| 1 | 0 | 1 | G416C | G377C | p.Gly416Cys | c.1246G>T | 10 | mild | - |
| 1 | 0 | 1 | Y244C | Y205C | p.Tyr244Cys | c.731A>G | 7 | mild | rs76026102 |
| 8 | 4 | 4 | L483P | L444P | p.Leu483Pro | c.1448T>C | 11 | severe | rs421016 |
| 7 | 4 | 3 | R159W | R120W | p.Arg159Trp | c.475C>T | 6 | severe | rs439898 |
| 3 | 1 | 2 | D448H | D409H | p.Asp448His | c.1342G>C | 10 | severe | rs1064651 |
| 2 | 2 | 0 | N421K | N382K | p.Asn421Lys | c.1263C>A | 10 | severe | - |
| 2 | 1 | 1 | G364R | G325R | p.Gly364Arg | c.1090G>A | 9 | severe | rs121908305 |
| 2 | 0 | 2 | R324H | R285H | p.Arg324His | c.971G>A | 8 | severe | rs79696831 |
| 1 | 1 | 0 | F252I | F213I | p.Phe252Ile | c.754T>A | 7 | severe | rs381737 |
| 1 | 1 | 0 | G241R | G202R | p.Gly241Arg | c.721G>A | 7 | severe | rs409652 |
| 1 | 1 | 0 | L422Pfs*3 | L383Pfs*3 | p.Leu422Profs*3 | c.1265_1319del | 10 | severe | rs80356768 |
| 1 | 1 | 0 | N227S | N188S | p.Asn227Ser | c.680A>G | 7 | severe | rs364897 |
| 1 | 1 | 0 | P161L | P122L | p.Pro161Leu | c.482C>T | 6 | severe | rs79637617 |
| 1 | 0 | 1 | L483R | L444R | p.Leu483Arg | c.1448T>G | 11 | severe | rs421016 |
| 1 | 0 | 1 | S146L | S107L | p.Ser146Leu | c.437C>T | 5 | severe | rs758447515 |
| 1 | 0 | 1 | E365K | E326K | p.Glu365Lys | c.1093G>A | 9 | risk | rs2230288 |
| 11 | 4 | 7 | R202Q | R163Q | p.Arg202Gln | c.605G>A | 7 | unknown | rs398123531 |
| 2 | 1 | 1 | L224R | L185R | p.Leu224Arg | c.671T>G | 7 | unknown | - |
| 2 | 0 | 2 | S310G | S271G | p.Ser310Gly | c.928A>G | 8 | unknown | rs1057942 |
| 2 | 0 | 2 | W387G | W348G | p.Trp387Gly | c.1159T>G | 9 | unknown | rs765182863 |
| 2 | 0 | 2 | W432C | W393C | p.Trp432Cys | c.1296G>T | 10 | unknown | - |
| 1 | 1 | 0 | L105Q | L66Q | p.Leu105Gln | c.314T>A | 5 | unknown | - |
| 1 | 1 | 0 | N231K | N192K | p.Asn231Lys | c.693T>A | 7 | unknown | - |
| 1 | 1 | 0 | Q536R | Q497R | p.Gln536Arg | c.1607A>G | 12 | unknown | rs750779755 |
| 1 | 1 | 0 | T107A | T68A | p.Thr107Ala | c.319A>G | 5 | unknown | - |
| 1 | 1 | 0 | T247I | T208I | p.Thr247Ile | c.740C>T | 7 | unknown | - |
| 1 | 1 | 0 | V34Cfs*56 | V(-6)Cfs*56 | p.Val34Cysfs*56 | c.100delG | 3 | unknown | - |
| 1 | 1 | 0 | V497E | V458E | p.Val497Glu | c.1490T>A | 11 | unknown | - |
| 1 | 0 | 1 | A33V | A(-7)V | p.Ala33Val | c.98C>T | 3 | unknown | rs776856496 |
| 1 | 0 | 1 | D419N | D380N | p.Asp419Asn | c.1255G>A | 10 | unknown | - |
| 1 | 0 | 1 | L363V | L324V | p.Leu363Val | c.1087C>G | 9 | unknown | rs1272814464 |
| 1 | 0 | 1 | P14P | P(-26)P | p.Pro14Pro | c.42T>G | 3 | unknown | rs1392613829 |
| 1 | 0 | 1 | P42P | P3P | p.Pro42Pro | c.126C>T | 4 | unknown | rs758949381 |
| 1 | 0 | 1 | R392Gfs*1 | R353Gfs*1 | p.Arg392Glyfs*1 | c.1174delC | 9 | unknown | - |
| 1 | 0 | 1 | S212S | S173S | p.Ser212Ser | c.636A>G | 7 | unknown | rs556008401 |
| 1 | 0 | 1 | S403S | S364S | p.Ser403Ser | c.1209C>T | 9 | unknown | rs773947710 |
| 1 | 0 | 1 | S52S | S13S | p.Ser52Ser | c.156G>A | 4 | unknown | rs756264143 |
| 1 | 0 | 1 | S77G | S38G | p.Ser77Gly | c.229A>G | 4 | unknown | - |
| 1 | 0 | 1 | V160V | V121V | p.Val160Val | c.480A>T | 6 | unknown | rs747409352 |
| 1 | 0 | 1 | V415V | V376V | p.Val415Val | c.1245C>T | 10 | unknown | rs755952419 |
| 3 | 3 | 0 | V499V | V460V | p.Val499Val | c.1497G>C | 11 | complex | rs1135675+ |
|  |  |  | A495P | A456P | p.Ala495Pro | c.1483G>C |  |  | rs368060+ |
|  |  |  | L483P | L444P | p.Leu483Pro | c.1448T>C |  |  | rs421016 |
| 2 | 2 | 0 | G241R | G202R | p.Gly241Arg | c.721G>A | 7 | complex | rs409652+ |
|  |  |  | S235P | S196P | p.Ser235Pro | c.703T>C |  |  | rs1064644+ |
|  |  |  | V230G | V191G | p.Val230Gly | c.689T>G |  |  | rs381427+ |
|  |  |  | N227K | N188K | p.Asn227Lys | c.681T>G |  |  | rs381418+ |
|  |  |  | N227S | N188S | p.Asn227Ser | c.680A>G |  |  | rs364897 |
| 1 | 1 | 0 | S16G | S(-24)G | p.Ser16Gly | c.46A>G | 3 | complex | rs1141804+ |
|  |  |  | L15S | L(-25)S | p.Leu15Ser | c.44T>C |  |  | rs1141802 |
| 1 | 0 | 1 | R202Q | R163Q | p.Arg202Gln | c.605G>A | 7-11 | complex | rs398123531+ |
|  |  |  | V499M | V460M | p.Val499Met | c.1495G>A |  |  | rs369068553 |
| 1 | 0 | 1 | S310G | S271G | p.Ser310Gly | c.928A>G | 8-11 | complex | rs1057942+ |
|  |  |  | P491P | P452P | p.Pro452Pro | c.1473C>T |  |  | rs149257166 |
| 1 | 0 | 1 | R202Q | R163Q | p.Arg202Gln | c.605G>A | 7-11 | complex | rs398123531+ |
|  |  |  | L483P | L444P | p.Leu483Pro | c.1448T>C |  |  | rs421016 |

*Note:* Allele name (HGVS) has been based on the reference sequence for *GBA* (NM_001005741) and the Human Genome Variation Society nomenclature (HGVS; http://varnomen.hgvs.org/). Allele name (traditional) is based on the common nomenclature, removing the first 39 amino acids.

Abbreviations: *GBA*-PD, *GBA*-related PD; PD, Parkinson's disease.

**Table S2.** NMSs classified by domain within the *GBA*-PD, early-iPD, and late-iPD groups

| **Variable** | ***GBA*-PD**  **n = 88** | **Early-iPD**  **n = 167** | **Late-iPD**  **n = 488** | ***p*** |
| --- | --- | --- | --- | --- |
| Cardiovascular | 45 (51.1) | 73 (43.7) | 223 (45.7) | 0.799 |
| Sleep | 77 (87.5) | 119 (71.3) | 398 (81.6) | 0.577 |
| Mood/cognitive | 67 (76.1) | 105 (62.9) | 332 (68.0) | 0.709 |
| Perception/hallucinations | 53 (60.2) | 63 (37.7) | 243 (49.8) | 0.883 |
| Attention/memory | 65 (73.9) | 111 (66.5) | 351 (71.9) | 0.576 |
| Gastrointestinal | 65 (73.9) | 99 (59.3) | 362 (74.2) | **0.038 ^a,b^** |
| Urinary | 52 (59.1) | 66 (39.5) | 285 (58.4) | **0.045 ^a,b^** |
| Sexual function | 23 (26.1) | 22 (13.2) | 90 (18.4) | 0.776 |
| Miscellaneous | 61 (69.3) | 109 (65.3) | 329 (67.4) | 0.578 |

*Note:* Data are provided as n (%). Groups were compared using logistic regression while adjusting for sex and disease duration.

Abbreviations: *GBA*-PD, *GBA*-related PD; early-iPD, early-onset iPD; iPD, idiopathic PD; late-iPD, late-onset iPD; NMSs, nonmotor symptoms; PD, Parkinson's disease.

^a^ Statistically significant differences between the *GBA*-PD and early-iPD subgroups.

^b^ Statistically significant differences between the late-iPD and early-iPD subgroups.

The values in **bold** are statistically significant differences (*p* < 0.05).
